# Cinobufagin Suppresses Melanoma Cell Growth by Inhibiting LEF1

**DOI:** 10.3390/ijms21186706

**Published:** 2020-09-13

**Authors:** Geon-Hee Kim, Xue-Quan Fang, Woo-Jin Lim, Jooho Park, Tae-Bong Kang, Ji Hyung Kim, Ji-Hong Lim

**Affiliations:** 1 Department of Biomedical Chemistry, College of Biomedical & Health Science, Konkuk University, Chungju 27478, Korea; rlarjsgml4@kku.ac.kr (G.-H.K.); gkrrnjs654852@kku.ac.kr (X.-Q.F.); lwj0908@kku.ac.kr (W.-J.L.); pkjhdn@kku.ac.kr (J.P.); 2 Diabetes and Bio-Research Center, Konkuk University, Chungju 27478, Korea; 3 Department of Biotechnology, College of Biomedical & Health Science, Konkuk University, Chungju 27478, Korea; kangtbko@kku.ac.kr; 4 Department of Biotechnology, College of Life Sciences and Biotechnology, Korea University, Seoul 02841, Korea

**Keywords:** LEF1, TCF4, cinobufagin, melanoma

## Abstract

Constitutive activation of the β-catenin dependent canonical Wnt signaling pathway, which enhances tumor growth and progression in multiple types of cancer, is commonly observed in melanoma. LEF1 activates β-catenin/TCF4 transcriptional activity, promoting tumor growth and progression. Although several reports have shown that LEF1 is highly expressed in melanoma, the functional role of LEF1 in melanoma growth is not fully understood. While A375, A2058, and G361 melanoma cells exhibit abnormally high LEF1 expression, lung cancer cells express lower LEF1 levels. A luciferase assay-based high throughput screening (HTS) with a natural compound library showed that cinobufagin suppressed β-catenin/TCF4 transcriptional activity by inhibiting LEF1 expression. Cinobufagin decreases LEF1 expression in a dose-dependent manner and Wnt/β-catenin target genes such as *Axin-2*, *cyclin D1*, and *c-Myc* in melanoma cell lines. Cinobufagin sensitively attenuates cell viability and induces apoptosis in LEF1 expressing melanoma cells compared to LEF1-low expressing lung cancer cells. In addition, ectopic LEF1 expression is sufficient to attenuate cinobufagin-induced apoptosis and cell growth retardation in melanoma cells. Thus, we suggest that cinobufagin is a potential anti-melanoma drug that suppresses tumor-promoting Wnt/β-catenin signaling via LEF1 inhibition.

## 1. Introduction

Malignant melanoma is the most dangerous type of skin cancer that develops from melanin-producing melanocytes [1]. The primary causes of melanoma are genetic mutations such as gain-of-function oncogene mutations (RAS, BRAF, ALK, and MET) and loss-of-function tumor suppressor mutations (TP53 and CDKN2A) that occur due to ultraviolet (UV) light exposure [2]. In addition, several cancer-promoting signaling pathways, including RAS-RAF-MEK-ERK, PI3K-AKT, and canonical Wnt/β-catenin signaling, promote malignant melanoma growth and progression [3]. In particular, recent studies have shown that Wnt signaling via the β-catenin/transcription factor 7-like 2 (TCF4)/Lymphoid enhancer-binding factor 1 (LEF1) complex activates transcriptional gene expression, including microphthalmia-associated transcription factor (MITF), tyrosinase-related protein-2 (TRP-2), tyrosinase (Tyr), and cyclin-dependent kinase 2 (Cdk2), which are associated with pigmentation, differentiation, and proliferation in melanocytes and malignant melanoma [4,5].

The canonical Wnt/β-catenin signaling pathway is associated with organ development, cellular proliferation, differentiation, and tissue homeostasis in multiple organisms [6]. In the absence of Wnt ligands, β-catenin interacts with α-catenin and localizes in the cytoplasmic membrane to maintain cell-to-cell interaction and communication [6]. β-catenin translocates from the cytoplasmic membrane to the nucleus upon Wnt ligand stimulation, and nuclear β-catenin is composed of the transcription factor complex with TCF4 (TCF7L2) that promotes development and proliferation via the expression of transcriptional genes such as Axin-2, CCND1, DKK1, and c-Myc [7]. The abnormal Wnt/β-catenin signaling pathway activation with increased TCF4 and LEF1 levels is also implicated in tumorigenesis and tumor progression in various cancer types [8,9,10]. Indeed, aberrant Wnt/β-catenin signaling pathway expression, which increases nuclear β-catenin, LEF1, and TCF4 levels, has been observed in various cancers, including melanoma [11,12,13].

LEF1 is a transcription factor that promotes the canonical Wnt/β-catenin signaling pathway by cooperating with TCF4 and is pathologically involved in cancer development and progression in multiple cancer types [14]. Previous studies have shown that ectopic LEF1 expression enhances the expression of epithelial-mesenchymal transition (EMT)-related genes, including the epithelial marker *E-cadherin* and mesenchymal markers *N-cadherin*, *vimentin*, *ZEB1*, and *SNAIL*, which are involved in cancer metastasis [15,16]. Thus, LEF1 is a promising molecular target for cancer treatment. Indeed, several studies have shown that LEF1 silencing attenuates cancer proliferation and induces apoptosis in glioblastoma multiforme (GBM) and colorectal cancer [17,18]. Furthermore, decreased cellular motility and invasiveness have been observed in *LEF1* knocked-down colorectal cancer cells [19]. Pharmacological suppression of LEF1 by selenite and ethacrynic acid (EA), which inhibit LEF1 transcriptional activity, was also found to attenuate cellular proliferation and promote cellular apoptosis in renal cell carcinoma, chronic lymphocytic leukemia, and multiple myeloma [20,21,22,23].

Although the number of clinical trials for the development of therapeutics including chemotherapy (cyclophosphamide), immunomodulating monoclonal antibodies therapies (anti-PD-1/PD-L1 and anti-CTLA4), and small molecule-targeted therapies (BRAF inhibitor) for melanoma treatment is increasing every year [24], canonical Wnt signaling-targeted therapy is not registered for melanoma therapy.

Here, we show that LEF1 is highly expressed in cultured melanoma cells and that melanoma cells expressing high levels of LEF1 are susceptible to LEF1 suppression. Furthermore, we performed a β-catenin/TCF4 binding promoter luciferase activity assay-based high throughput screening using a natural products library, containing 502 single compounds isolated from Chinese herbs, to find an inhibitor of β-catenin/TCF4/LEF1-mediated cancer growth and progression. In this screening, we found that the natural compound, cinobufagin, was a potent inhibitor of canonical Wnt/β-catenin signaling, decreasing LEF1 expression and consequently suppressing melanoma growth and inducing apoptosis. These results suggest that cinobufagin is a potential LEF1 inhibitor and a promising molecular marker for the development of targeted cancer therapy, especially for melanoma treatment.

## 2. Results

### 2.1. LEF1 Suppression Decreases Melanoma Cell Viability

**Figure 1:**
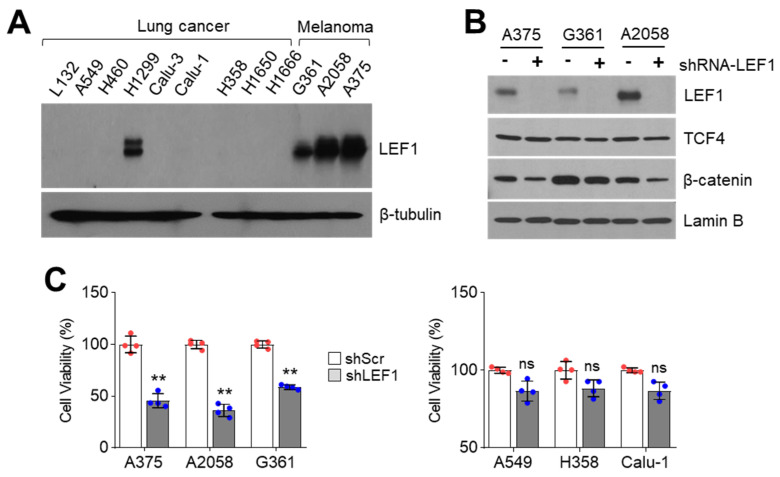
Suppression of LEF1 attenuates melanoma cell growth. (**A**) LEF1 expression in lung cancer and melanoma cell lines. LEF1 protein levels were measured by using Western blotting. (**B**) LEF1 knock-down efficiency in melanoma cell lines. A375, A2058, and G361 melanoma cell lines were infected with lentivirus expressing shRNA against control or LEF1. The infected cells were selected by puromycin for six days. −, shRNA-scramble and +, shRNA-LEF1. (**C**) LEF1 knock-down attenuates melanoma cell growth but not lung cancer cells. LEF1 knocked-down melanoma and lung cancer cells were generated and cell viability was measured by crystal violet staining. The values represent the mean ± SD (*n* = 4); ns, not significant and ** *p* < 0.01. Red and blue points, mean value of individual sample.

Emerging evidence has shown that Lymphoid enhancer-binding factor 1 (LEF1) expression is highly increased in leukemia and various types of solid cancer, such as lung adenocarcinoma, colorectal cancer, prostate cancer, and malignant melanoma [14]. Although it is well known that LEF1 usually involves melanocyte differentiation and phenotype switching by increasing microphthalmia-associated transcription factor (MITF), the pro-survival role of LEF1 in melanoma is not fully understood [4,5]. Thus, we attempted to determine whether LEF1 acts as a pro-survival factor for melanoma cell growth. Initially, nuclear LEF1 levels in nine lung cancer cells and three melanoma cells were analyzed. Surprisingly, highly increased nuclear LEF1 levels were observed in A375, A2058, and G361 melanoma cells, but increased nuclear LEF1 was only detected in H1299 cells among nine lung cancer cell lines (Figure 1A). We generated LEF1 silenced melanoma cells using short hairpin RNA (shRNA) to understand the functional role of LEF1 on melanoma cell growth and observed decreased LEF1 protein levels in LEF1 knocked-down A375, A2058, and G361 cells (Figure 1B). In addition, decreased β-catenin but not TCF4 levels were observed in LEF1 knocked-down melanoma cells (Figure 1B). LEF1 suppression attenuated cell viability in LEF1-high expressing melanoma cells but not LEF1-low expressing A549, H358, and Calu-1 lung cancer cells (Figure 1C). These results suggest that LEF1 is predominantly expressed in melanoma rather than lung cancer cells and that LEF1-high expressing melanoma cells are susceptible to suppression of cell growth caused by LEF1 inhibition.

### 2.2. Identification of Cinobufagin as a Wnt/β-Catenin Signaling Pathway Inhibitor

**Figure 2:**
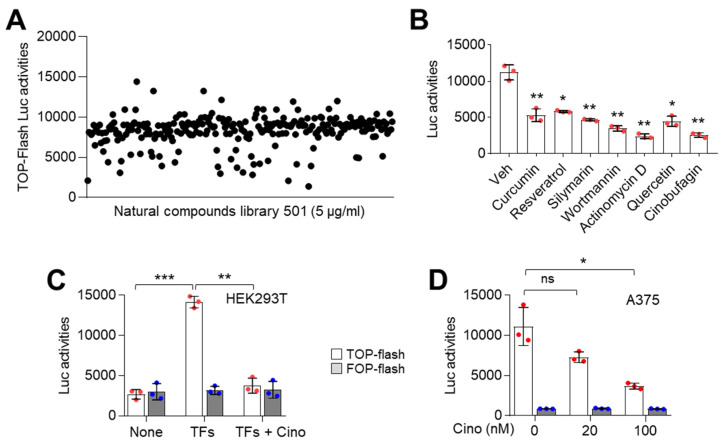
TOP-flash luciferase assay-based high throughput screening for β-catenin/TCF4 inhibiting natural compounds. (**A**) TOP-flash luciferase activities were suppressed by several natural compounds. HEK293T cells were transiently co-transfected with TOP-flash luciferase, pcDNA/Myc-TCF4, pBABE-puro-LEF1, and pcDNA3-β-catenin. Transfected cells were incubated for 24 h with natural product library 502 as described in the Materials and Methods section. Hit compounds were selected by following decreased fold change three times compared to DMSO control. (**B**) Hit compounds inhibit TOP-flash luciferase activities in A375 melanoma cells. TOP-flash luciferase plasmids transfected A375 cells were incubated for 24 h with 5 μM of hit compounds as indicated. The values represent the mean ± SD (*n* = 3); * *p* < 0.05 and ** *p* < 0.01. (**C**) Cinobufagin as a potent inhibitor of β-catenin/TCF4 suppresses TOP-flash luciferase activities. HEK293T cells were transfected with transcription factors (TFs) including pcDNA/Myc-TCF4, pBABE-puro-LEF1, and pcDNA3-β-catenin which activates β-catenin/TCF4 signaling. Transfected HEK293T cells were incubated for 24 h with 5 μM of cinobufagin. The values represent the mean ± SD (*n* = 3); ** *p* < 0.01 and *** *p* < 0.001. (**D**) Cinobufagin inhibits β-catenin/TCF4 transcriptional activities in A375 melanoma cells in a dose-dependent manner. A375 cells were transfected with TOP or FOP-flash luciferase plasmids. Transfected A375 cells were incubated for 24 h with 20 or 100 nM of cinobufagin. The values represent the mean ± SD (*n* = 3); * *p* < 0.05. Red and blue points, mean value of individual sample.

Abnormal activation of the canonical Wnt/β-catenin signaling pathway is observed in various cancers and is associated with cancer progression [11,12,13]. In addition, we found that LEF1, an essential transcriptional component of Wnt/β-catenin signaling, is aberrantly expressed in melanoma cells and involved in cell growth (Figure 1). Thus, we performed high throughput screening (HTS) using a natural product library containing 502 single compounds to identify a potential Wnt/β-catenin signaling pathway inhibitor. In this screening, we used the TOP-flash reporter plasmid containing three copies of the T-cell factor (TCF) binding site. Initially, HEK293 cells were transfected with plasmids encoding β-catenin, TCF4, and LEF1 with TOP-flash reporter and incubated with 5 μg/mL of a single compound contained in the natural products library. Using luciferase activity analysis, we found seven single compounds: curcumin, resveratrol, silymarin, wortmannin, actinomycin D, quercetin, and cinobufagin; these compounds resulted in a three-fold decrease of TOP-flash reporter activity (Figure 2A,B). Since the suppressive role of cinobufagin on Wnt/β-catenin signaling has not been investigated, we selected cinobufagin as a potential Wnt/β-catenin signaling inhibitor. To confirm whether cinobufagin inhibits TOP-flash reporter activity by suppressing the thymidine kinase promoter, we further analyzed the effect of cinobufagin on the activity of FOP-flash reporter with mutated TCF binding site, followed by three copies in the reverse orientation of TCF binding sequences. As expected, the FOP-flash reporter activity was not affected by cinobufagin (Figure 2C). Abnormally increased levels of nuclear LEF1, TCF4, and β-catenin, which are essential transcriptional components of Wnt/β-catenin signaling, were observed in A375, A2058, and G361 melanoma cells (Figure 1B). We further examined whether cinobufagin inhibits TOP-flash reporter activity in Wnt/β-catenin signaling activated A375 melanoma cells. Consistently, cinobufagin significantly suppressed TOP-flash reporter activity induced by endogenous nuclear LEF1, TCF4, and β-catenin (Figure 2D). These results suggest that cinobufagin may inhibit transcription factors regulated by canonical Wnt/β-catenin signaling.

### 2.3. Cinobufagin Decreases LEF1 Expression

**Figure 3:**
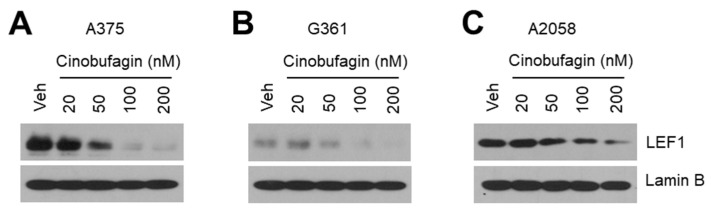
Cinobufagin decreases LEF1 expression in melanoma cells. (**A**–**C**) Cinobufagin suppresses nuclear LEF1 protein levels in a dose-dependent manner in A375 (**A**), G361 (**B**), and A2058 (**C**) melanoma cells. Cells were incubated for 24 h with different concentrations of cinobufagin as indicated. After cinobufagin treatment, nuclear protein was isolated and protein levels were measured by Western blotting.

To elucidate the regulatory mechanism by which suppression of TOP-flash reporter activity by cinobufagin treatment, we examined whether cinobufagin suppresses the expression of LEF1—a transcription factor activating Wnt/β-catenin signaling in melanoma cells. Interestingly, we found that cinobufagin decreased nuclear LEF1 levels in a dose-dependent manner in A375 (Figure 3A), G361 (Figure 3B), and A2058 (Figure 3C) melanoma cells, indicating that cinobufagin may attenuate canonical Wnt/β-catenin signaling by decreasing LEF1 expression in melanoma.

### 2.4. Cinobufagin Suppresses Downstream Wnt/β-Catenin Signaling Target Genes Dependent on LEF1 Expression

**Figure 4:**
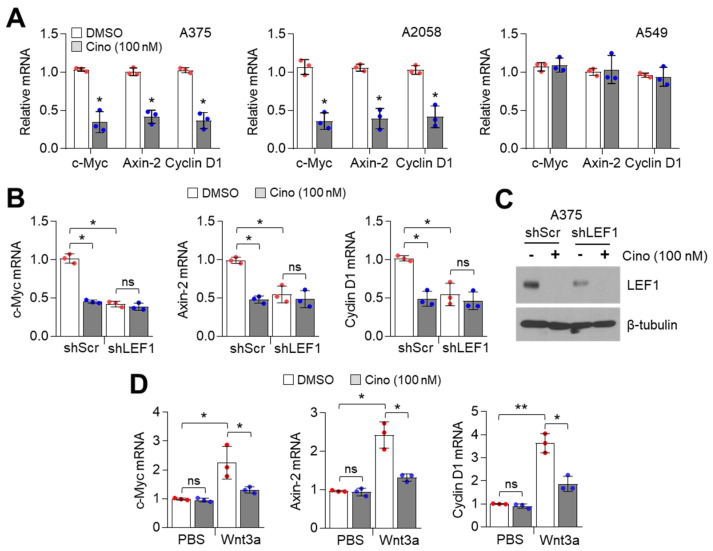
Cinobufagin suppresses expression of β-catenin/TCF4-induced target genes in melanoma cells. (**A**) β-catenin/TCF4 target genes decreased by cinobufagin in LEF1-high expressing melanoma cells, but not LEF1-low expressing A549 lung cancer cells. Cells were incubated with cinobufagin for 24 h. After cinobufagin treatment, gene expression levels were measured by qRT-PCR. The values represent the mean ± SD (*n* = 3); * *p* < 0.05. (**B**) β-catenin/TCF4 target genes are not responsive to cinobufagin in LEF1 knocked-down A375 cells. Cinobufagin was treated for 24 h in LEF1 knocked-down A375 cells. β-catenin/TCF4 target genes expression levels were measured by qRT-PCR. The values represent the mean ± SD (*n* = 3); * *p* < 0.05 and ns, not significant. (**C**) LEF1 knock-down efficiency in A375 cells. LEF1 protein levels were measured by Western blotting. (**D**) Wnt3a-induced β-catenin/TCF4 target genes are decreased by cinobufagin in human melanocytes. Recombinant Wnt3a protein was dissolved in 1X PBS containing 0.1% endotoxin-free HSA. Wnt3a (30 ng/mL) pretreated for 1h prior to cinobufagin treatment, and then cells were incubated for 24 h. β-catenin/TCF4 target genes expression levels were measured by qRT-PCR. The values represent the mean ± SD (*n* = 3); * *p* < 0.05 and ** *p* < 0.01. ns, not significant. Red and blue points, mean value of individual sample.

LEF1 increases various downstream target genes to regulate cell proliferation, cell cycle progression, anti-apoptosis, and EMT by cooperating with β-catenin and TCF4 in response to Wnt signaling [25]. We examined whether downstream target genes of Wnt/β-catenin signaling are inhibited by cinobufagin in melanoma cells and found that 100 nM of cinobufagin significantly decreased *c-Myc*, *Cyclin D1*, and *Axin-2* mRNA levels in LEF1-high expressing melanoma (A375 and A2058) but not in LEF1-low expressing lung cancer (A549) cells (Figure 4A). To study whether LEF1 is required to suppress the expression of Wnt/β-catenin target genes, we analyzed *c-Myc*, *Cyclin D1*, and *Axin-2* mRNA levels in LEF1 knocked-down A375 melanoma cells. Figure 4B shows that cinobufagin-mediated suppression of Wnt/β-catenin target genes was abolished in LEF1 knocked-down A375 melanoma cells. LEF1 knock-down efficiency was confirmed (Figure 4C). In addition, cinobufagin suppressed Wnt3a-induced canonical Wnt signaling-related genes expression in human melanocytes (Figure 4D). These results indicate that cinobufagin inhibits downstream target genes of canonical Wnt/β-catenin by suppressing LEF1 in melanoma cells.

### 2.5. Cinobufagin Induces Apoptosis by Suppressing LEF1 in Melanoma Cells

**Figure 5:**
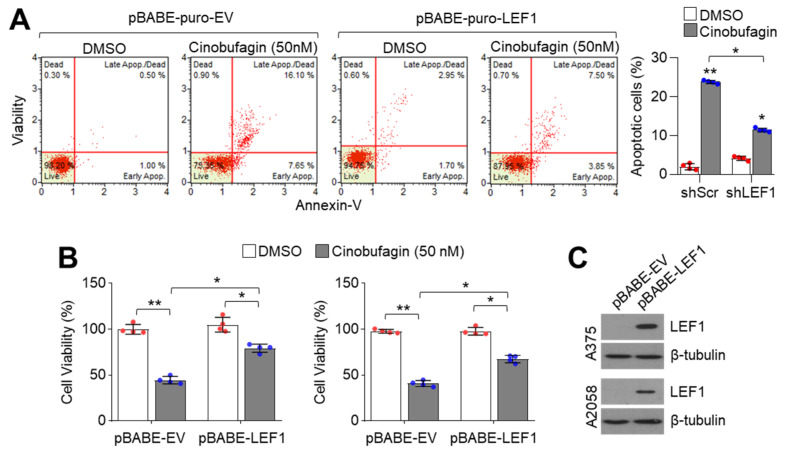
Cinobufagin suppresses melanoma cell growth by suppressing LEF1. (**A**) Cinobufagin induces apoptosis. LEF1 stably expressing A375 melanoma cells were incubated with cinobufagin for 72 h. The apoptotic cell population was measured by Annexin-V staining as described in the Materials and Methods section. (**B**) Cinobufagin attenuates melanoma cell growth dependent on LEF1 expression. LEF1 overexpressing A375 and A2058 melanoma cells were incubated with cinobufagin for 72 h. Cell viability was measured by crystal violet staining. The values represent the mean ± SD (*n* = 4); * *p* < 0.05 and ** *p* < 0.01. (**C**) LEF1 expression in pBABE-EV or pBABE-LEF1 expressing A375 and A2058 cells. LEF1 protein levels were measured by Western blotting. Red and blue points, mean value of individual sample.

LEF1 suppression decreased viability in melanoma but not lung cancer cells (Figure 1B), indicating that LEF1 acts as a pro-survival factor at least in LEF1-high expressing melanoma cells. Thus, we examined the pro-apoptotic effect of cinobufagin and observed increased apoptotic cell numbers in 50 nM of cinobufagin-treated A375 melanoma cells (Figure 5A). Moreover, cinobufagin-induced apoptosis was abolished in ectopic LEF1 expression (Figure 5A). Consistently, decreased cell viability in cinobufagin-treated A375 and A2058 melanoma cells was reversed by ectopic LEF1 expression (Figure 5B). Overexpressed LEF1 levels were confirmed in Figure 5C. These results indicate that cinobufagin induces apoptosis by decreasing LEF1 expression in melanoma cells.

## 3. Discussion

Increasing evidence has shown that LEF1, which belongs to the T cell factor (TCF) family, acts as a cancer-promoting transcription factor by cooperating with the canonical Wnt/β-catenin signaling pathway [14]. Thus, LEF1 is considered a potential therapeutic target for cancer treatment. LEF1 knock-down and suppression using shRNA and small molecules decreased cell growth in high LEF1 expressing cancer cells [17,18]. In this study, we found that LEF1 is highly expressed in A375, A2058, and G361 melanoma but not various lung cancer cells. Consistent with previous studies, we found that activated Wnt/β-catenin signaling, which involves LEF1, TCF4, and β-catenin is highly increased in melanoma [10]. Moreover, elevated nuclear β-catenin levels were also observed in human melanoma specimens [26], indicating that activated canonical Wnt/β-catenin signaling has an important role in melanoma and melanocyte physiology. Activated canonical Wnt/β-catenin signaling was found to be associated with decreased melanoma proliferation, suggesting that Wnt/β-catenin signaling activation is a negative regulator of melanoma growth in patient-derived tumor tissues and mouse melanoma models [27]. Additionally, it has been reported that the differential expression of LEF1 and TCF4 determines the proliferative and invasive potentials of melanoma subtypes [10]. Predominant expression levels of LEF1 and β-catenin, but not TCF4, have been observed in highly metastatic melanoma cells such as MM-AN, MM-BP, RPM-EP, and MM-RU cells [28]. Although previous studies indicate that Wnt/β-catenin signaling with LEF1, TCF4, and β-catenin expression is closely associated with melanoma growth and progression, it is still unclear whether LEF1 acts as a proliferative factor in melanoma. Here, we used a cell viability assay to understand whether LEF1 expression is associated with proliferative potential in cultured melanoma cells. Our study revealed that LEF1 suppression induced cell growth retardation and apoptosis in LEF1-high expressing melanoma cells but not in LEF1-low expressing A549 lung cancer cells. These results suggest that LEF1-high expressing cells may be susceptible to LEF1-induced inhibition of proliferation.

Several small molecules have been found to exert anti-cancer effects by suppressing LEF1 and Wnt/β-catenin signaling in multiple cancer types. The combination of the DNA methyltransferase inhibitor 5-aza-2′-deoxycytidine (DAC) and the chemotherapeutic agent paclitaxel was found to enhance LEF1 suppression and attenuate tumor growth in renal cell carcinoma (RCC) cells with high levels of LEF1 expression [29]. Natural pharmacological products with effective antioxidant, anti-infective, anti-inflammatory, anti-angiogenic, and anti-carcinogenic properties have been used to treat various human diseases, such as cancers and cardiovascular diseases [30]. Thus, we attempted to find natural compounds that target Wnt/β-catenin signaling, which may be potential therapeutics for cancer treatment. Here, we performed high throughput screening using a natural product library consisting of 502 single compounds and found that curcumin, resveratrol, silymarin, quercetin, and cinobufagin inhibit TOP-flash luciferase activity induced by β-catenin/TCF4/LEF1. Consistent with our screening, Tiwari et al. showed a protective role of curcumin via the suppression of canonical Wnt/β-catenin signaling in Bisphenol-A mediated inhibition of hippocampal neurogenesis [31]. The suppressive effect of apigenin, curcumin, and ursolic acid via inhibition of Wnt/β-catenin signaling on colorectal cancer cell proliferation has also been observed [32,33,34]. A recent report showed that LEF1 and multidrug resistance-related genes, such as *ABCG2*, *VIM*, and *Cav1*, are highly increased in docetaxel (DTX)-resistant MCF7 breast cancer cells, indicating that LEF1 is involved in multidrug resistance in cancer [35]. Moreover, in vitro analysis has shown that quercetin reversed DTX resistance and induced apoptosis by suppressing LEF1 expression in DTX-resistant MCF7 breast cancer cells [35]. Here, we demonstrate that cinobufagin has an anti-melanoma effect by decreasing LEF1 and Wnt/β-catenin signaling.

Cinobufagin, which was approved by the Chinese State Food and Drug Administration as a chemotherapeutic drug for liver and prostate cancer treatment in China, is a major active ingredient isolated from the Traditional Chinese Medicine Venenum Bufonis [36]. Although cytotoxic side effects in cardiac myocytes by the administration of a high dose of cinobufagin contained in Chinese Medicine has been reported [37], emerging evidence has shown that cinobufagin effectively suppresses cancer growth and progression by inducing cell cycle arrest and apoptosis and inhibiting angiogenesis [38]. For example, cinobufagin was found to promote cell cycle arrest at the G2/M phase and apoptosis in hepatocellular carcinoma and esophageal squamous cell carcinoma by increasing the expression levels of pro-apoptotic p73 and BAX [39,40]. Cinobufagin was found to induce apoptosis in osteosarcoma cancer cells by suppressing cancer-promoting signaling pathways such as STAT3, Notch and NF-kB, and activating mitochondria-mediated apoptosis pathways [41,42,43,44]. The anti-angiogenic effect of cinobufagin by suppressing the mammalian target of rapamycin (mTOR) and hypoxia-inducible factor-1α (HIF1α) signaling pathways has also been observed in colorectal cancer [45].

Here, our results show that cinobufagin has an anti-cancer effect by inhibiting LEF1-mediated activation of Wnt/β-catenin signaling, especially in LEF1 high expressing melanoma cells, indicating that cinobufagin is a promising natural pharmaceutical agent for melanoma treatment. Cinobufagin was found to decrease LEF1 expression in A375, A2058, and G361 melanoma cells in a dose-dependent manner. In line with this observation, the expressions of cancer-promoting genes like *c-Myc*, *cyclin D1*, and *Axin-2*, which are regulated by LEF1 and Wnt/β-catenin signaling, were significantly decreased by cinobufagin. Interestingly, the suppressive effect of cinobufagin on LEF1 and Wnt/β-catenin downstream target gene expression was diminished by LEF1 knock-down in A375 melanoma cells, suggesting that LEF1 is a potential molecular target of cinobufagin.

Despite the emerging evidence that cinobufagin has an anti-cancer effect in various cancers, the regulatory molecular mechanism by which cinobufagin attenuates cancer cell proliferation and induces apoptosis is not clear. Here, the major finding is that cinobufagin suppresses melanoma cell growth and induces apoptosis by inhibiting the LEF1-mediated Wnt/β-catenin signaling pathway. Taken together, our results demonstrate that cinobufagin is a promising anti-cancer drug that may be useful for developing targeted therapeutics in LEF1-high expressing cancers.

## 4. Materials and Methods

### 4.1. Reagents and Antibodies

The natural compound library contains 502 single compounds was obtained from Enzo Biochem (Farmingdale, NY, USA). Cinobufagin (C1272), Curcumin (C1386), Resveratrol (R5010), Silymarin (S0292), Wortmannin (W1628), Actinomycin D (A1410), Quercetin (Q4951), and human Wnt3a recombinant protein (H17001) expressed in HEK293 cells were purchased from Sigma Aldrich (St. Louis, MO, USA). Antibodies recognizing LEF1 (CST-2230), TCF4 (CST-2569), β-catenin (CST-8480), Lamin B (sc-374015), and β-tubulin (sc-9104) were purchased from Cell Signaling Technology (Danvers, MA, USA) and Santa Cruz Biotechnology (Dallas, TX, USA). A lyophilized powder form of cinobufagin was dissolved in dimethyl sulfoxide (DMSO) for 0.1 mM stock, and then cinobufagin was used to treat cells at 20–200 nM for 12–72 h.

### 4.2. Cell Culture and Cell Viability Assay

Lung cancer (A549, H460, H1299, Calu-3, Calu-1, H358, H1650, and H1666) and melanoma cell lines (G361, A375, and A2058) were obtained from the Korean Cell Line Bank (Seoul, Korea) and American Type Culture Collection (Manassas, VA, USA) and cultured in Dulbecco’s modified Eagle’s medium (DMEM) and Roswell Park Memorial Institute medium (RPMI1640) supplemented with 10% fetal bovine serum. Cell viability was measured by using a crystal violet assay. In brief, A375, A2058, and G361 melanoma cells were infected with lentivirus expressing short hairpin RNA (shRNA) against human LEF1 or scramble. After 24 h incubation, infected cells were selected with puromycin for 6 days, and then cell viability was measured. To measure cell viability in the absence or presence of cinobufagin in LEF1 overexpressing A375 and A2058 cells, cells were transiently transfected with pBABE-puro-LEF1 or pBABE-puro-empty vector (EV) by using Lipofectamine 2000. Transfected cells were incubated for 48 h to allow stabilization and enough protein expression. After incubation, 100 nM of cinobufagin was treated for 48 h. To measure cell viability, the cells were seeded into 12- or 24-well tissue culture dishes, and then cells were washed and fixed with phosphate-buffered saline (PBS) and 4% paraformaldehyde. The cells were stained with 0.5% crystal violet solution at room temperature for 20 min, and the stained cells were solubilized in 1% sodium dodecyl sulfate (SDS) solution. After fixation and staining, the optical density was measured at 570 nm by using an absorbance reader (BioTek, Winooski, VT, USA) (OD570).

### 4.3. Plasmids

pBABE-puro LEF1 (Addgene plasmid # 27023) [46], pcDNA/Myc-TCF4 (Addgene plasmid # 16512) [47] and pcDNA3-β-catenin (Addgene plasmid # 16828) [48] were a gift from Joan Massague, Bert Vogelstein and Eric Fearon, respectively. pBABE-puro (Addgene plasmid # 1764) [49] were a gift from Hartmut Land, Jay Morgenstern, and Robert Weinberg. Human LEF1 targeting pLKO.1-shRNA (TRCN0000020162) was obtained from Sigma Aldrich (St. Louis, MO, USA). TOP or FOP-flash reporter plasmids were used to measure β-catenin/TCF4 transcriptional activity [50].

### 4.4. Western Blotting

Western blotting was performed to examine the alteration of protein expression. Total proteins were isolated from cultured cells by lysis buffer (1% IGEPAL, 150 mM NaCl, 50 mM Tris-HCl (pH 7.9), 10 mM NaF, 0.1 mM EDTA, and a protease inhibitor cocktail). Total proteins were subjected to sodium dodecyl sulfate (SDS)-polyacrylamide gel electrophoresis (PAGE) to separate the proteins by molecular weight. After SDS-PAGE, the electrophoresed proteins were then transferred onto polyvinylidene fluoride (PVDF) membranes (Millipore, Burlington, MA, USA). The membranes with transferred proteins were incubated with primary antibodies (1:1000–1:5000 dilution) diluted in 5% skim milk for 24 h at 4 °C, and then horseradish peroxidase (HRP)-conjugated secondary antibodies (1:10,000) for 1 h at room temperature, respectively. The protein expression levels were visualized by using the Enhanced Chemiluminescence (ECL) Prime kit (GE Healthcare, Pittsburgh, PA, USA).

### 4.5. Quantitative Real-Time PCR

To measure mRNA expression, quantitative real-time PCR was performed as described previously [51]. Briefly, total RNA was isolated from cultured cells by using TRIzol reagent (Invitrogen, Carlsbad, CA, USA) and 2 μg of total RNA was used for cDNA synthesis by using a high-capacity cDNA reverse transcription kit (Applied Biosystems, Waltham, MA, USA). The human 36B4 (rplp0, acidic ribosomal phosphoprotein P0) gene was used for housekeeping control. The SYBR Green PCR Master Mix (Applied Biosystems, Waltham, MA, USA) was used for quantitative PCR. The primer sequences used in the experiment are shown in Table 1.

### 4.6. Apoptosis Assays

The apoptotic cell population was measured by using Muse™ Annexin V and Dead Cell kit (Millipore, Burlington, MA, USA) in accordance with a previously described [51]. Initially, LEF1 stably overexpressing A375 and A2058 cells were generated with puromycin selection. Empty vector (pBABE-puro-empty vector) or LEF1 (pBABE-puro-LEF1) overexpressing A375 and A2058 cells (1 × 10^5^ cells/well) were seeded onto a 6-well cell culture plate and incubated for 48 h in the absence or presence of 100 nM of cinobufagin. After cinobufagin treatment, the cells were harvested and washed with cold PBS, and then cells were incubated in 100 μL Muse™ Annexin V and Dead Cell kit reagents (Millipore, Burlington, MA, USA) for 20 min at room temperature. Finally, the cells were subjected to Mini Flow Cytometry Muse™ Cell Analyzer (Millipore, Burlington, MA, USA) to measure apoptotic cell populations.

### 4.7. Luciferase Assay

To screen single compounds derived from natural products which attenuate β-catenin/TCF4 transcriptional activity, TOP and FOP flash luciferase plasmids (100 ng/mL) were transiently transfected into HEK293T cells with ectopic expression of pBABE-puro-LEF1 (1 μg/mL), pcDNA-Myc-TCF4 (0.5 μg/mL), and pcDNA3-β-catenin (0.5 μg/mL). The transfected HEK293T cells were seeded in 96-well culture dishes and incubated for 24 h to allow stabilization and protein expression. After incubation, cells were incubated with a natural compound library containing 502 single compounds at 5 μM of final concentration for 24 h. To measure β-catenin/TCF4 transcriptional activity in A375 melanoma cells, TOP and FOP flash luciferase plasmids (0.5 μg/mL) were transiently transfected using Lipofectamine 2000 without co-transfection of pBABE-puro-LEF1, pcDNA-Myc-TCF4, and pcDNA3-β-catenin. Luciferase activities were analyzed by using a Luciferase assay system (Promega, Madison, WI, USA) and Synergy 2 Luminometer (BioTek, Winooski, VT, USA), and then luciferase activities were normalized to the activity of β-galactosidase.

### 4.8. Statistical Analysis

The unpaired Student’s t-test was performed for statistical analysis. The data are presented as the mean ± standard deviation (SD). A *p*-value of < 0.05 was considered statistically significant.

## Figures and Tables

**Table 1:** Primer sequences for quantitative real time-PCR.

Gene	Forward Primer	Reverse Primer
c-Myc	5′-CGTCTCCACACATCAGCACAA-3′	5′-CACTGTCCAACTTGACCCTCTTG-3′
Axin-2	5′-GAGTGGACTTGTGCCGACTTCA-3′	5′-GGTGGCTGGTGCAAAGACATAG-3′
Cyclin D1	5′-GGCGGAGGAGAACAAACAGA-3′	5′-TGGCACAAGAGGCAACGA-3′
36B4	5′-CATGTTGCTGGCCAATAAGG-3′	5′-TGGTGATACCTAAAGCCTGGAA-3′
